# Altered likelihood of brain activation in attention and working memory networks in patients with multiple sclerosis: An ALE meta-analysis^☆^

**DOI:** 10.1016/j.neubiorev.2013.09.005

**Published:** 2013-12

**Authors:** K. Kollndorfer, J. Krajnik, R. Woitek, J. Freiherr, D. Prayer, V. Schöpf

**Affiliations:** a Department of Biomedical Imaging and Image-Guided Therapy, Medical University of Vienna, Austria; b Department of Neurosurgery, Medical University of Vienna, Austria; c Diagnostic and Interventional Neuroradiology, RWTH Aachen University, Aachen, Germany

**Keywords:** Multiple sclerosis, Brain imaging, Working memory, Attention, Activation likelihood estimation, VLPFC, DLPFC, n-Back, PASAT, PVSAT

## Abstract

Multiple sclerosis (MS) is a chronic neurological disease, frequently affecting attention and working memory functions. Functional imaging studies investigating those functions in MS patients are hard to compare, as they include heterogeneous patient groups and use different paradigms for cognitive testing. The aim of this study was to investigate alterations in neuronal activation between MS patients and healthy controls performing attention and working memory tasks. Two meta-analyses of previously published fMRI studies investigating attention and working memory were conducted for MS patients and healthy controls, respectively. Resulting maps were contrasted to compare brain activation in patients and healthy controls. Significantly increased brain activation in the inferior parietal lobule and the dorsolateral prefrontal cortex was detected for healthy controls. In contrast, higher neuronal activation in MS patients was obtained in the left ventrolateral prefrontal cortex and the right premotor area. With this meta-analytic approach previous results of investigations examining cognitive function using fMRI are summarized and compared. Therefore a more general view on cognitive dysfunction in this heterogeneous disease is enabled.

## Highlights

• We conducted an ALE meta-analysis of fMRI studies investigating MS patients.
• We included nine fMRI studies performing working memory/attention tasks.
• Healthy controls showed higher activation in the IPL and the DLPFC.
• For MS patients higher activation was obtained in the VLPFC.

## 1 Introduction

Multiple Sclerosis (MS) is an inflammatory and neurodegenerative disease of the central nervous system (CNS) characterized predominantly by demyelinating lesions in the white matter of the brain and the spinal cord. Conventional structural magnetic resonance imaging (MRI) can be used to identify and quantify these lesions. Furthermore, focal demyelination and neuronal loss of gray matter, appearing as partly or entirely cortically located lesions on MRI images as well as structural damage of white and gray matter appearing normal on conventional MRI images are components of the disease (Lassmann, 2008). A hallmark of CNS lesions characteristic for MS is disseminations in both space and time. Due to spatially disseminated damage to the CNS, MS results in a wide spectrum of clinical manifestations ranging from motor symptoms to cognitive and neuropsychiatric deficits. Disease onset peaks between 22 and 30 years and women are affected approximately twice as often as men (Alonso and Hernán, 2008).

The different clinical courses of MS can be categorized into four types based on disease progression (Lublin and Reingold, 1996): Relapsing-remitting MS (RRMS) which is characterized by clearly defined relapses with full recovery or sequelae and residual defects. During periods between relapses the disease does not progress clinically. In case this phenotype of the disease is followed by a progression with or without occasional relapses, minor remissions, and plateaus it is classified as secondary progressive MS (SPMS). In contrast, primary progressive MS (PPMS) takes a progressive course from the beginning with plateaus and temporary minor improvements. The fourth type is progressive-relapsing MS (PRMS), which is progressive from the onset with acute relapses. Between the relapses there is continuing progression. Superimposed relapses may occur in SPMS, whereas in PPMS no acute relapses occur (patients with relapses are then categorized as having PRMS; Lublin and Reingold, 1996).

Among the clinical symptoms which affect all types of MS cognitive impairment is the most common symptom with prevalence rates between 43% and 70% significantly contributing to the extent of disability (Benedict et al., 2006; Peyser et al., 1990; Rao et al., 1991). Memory, attention, processing speed, information processing efficiency, and executive functioning have been shown to be the cognitive capacities that are most frequently impaired (Benedict et al., 2006; Rao et al., 1991).

Functional MRI (fMRI) has been used to identify brain regions that are on the one hand involved in cognitive functioning in healthy individuals and on the other hand showing altered activation in MS. FMRI studies that explored cognitive processes in MS examined a great variety of functions, such as working memory, attention, and executive functions (Chiaravalloti and DeLuca, 2008) using paradigms such as the Paced Auditory Serial Addition Test (PASAT; e.g. Audoin et al., 2005; Forn et al., 2006; Mainero et al., 2004), the Paced Visual Serial Addition Test (PVSAT; Bonzano et al., 2009), and the n-Back task (e.g. Amann et al., 2011; Cader et al., 2006; Forn et al., 2007; Sweet et al., 2004). These abilities were not only examined in behavioral studies, but also using functional imaging to explore the neuronal correlates of impaired performance.

During the last years, the number of functional imaging studies rapidly increased as the neuroscience community urged to gain more detailed insight into diseases progression and prognosis, as well as therapeutic options. However, results of these studies are hardly comparable, as typically stimulation paradigms, disease phenotypes, and statistical evaluation of fMRI data show huge variability. Therefore, the current study aimed at providing an overview of previous literature in conjunction with the mapping of functional brain activity related to attention and working memory function in MS patients with high statistical probability performing meta-analyses in order to present a comparison of neuronal activity patterns of MS patients with those of healthy controls.

## 2 Materials and methods

### 2.1 Study selection

For this meta-analysis peer-reviewed studies on functional neuroimaging of attention and working memory processes in patients with multiple sclerosis, published in the English language between 1996 and February 2013 were identified.

Literature research was performed using PubMed, an online database including more than 22 million citations for biomedical literature using the following keywords: *functional MRI*; *positron emission tomography*; *multiple sclerosis* (including common abbreviations like *fMRI*, *PET*, and *MS*); which were cross-referenced with the search terms *cognition*; *information processing speed*; *memory*; *working memory*; *executive functions*; *selective; focused or sustained attention*; and *attention*. In addition, we used search terms for tasks associated with working memory and attention like n-Back; *Paced Auditory Serial Addition Test*; and *Paced Visual Serial Addition Test* (including the common acronyms PASAT and PVSAT) as cross-reference. In a second step, the reference lists of the original articles resulting from this search were examined in order to find additional publications that were not identified by the database search.

For the current meta-analysis the following seven inclusion criteria were specified:

1. Studies must include patients with diagnosed multiple sclerosis, studies including patients with Clinically Isolated Syndrome (CIS) with the diagnosis “possible MS” were excluded.
2. Included studies had to focus on attention and working memory processes by using auditory or visually presented stimuli. Studies, that used cognitive paradigms investigating attention in conjunction with higher cognitive abilities, such as response inhibition, were excluded.
3. The studies had to examine neuronal activity in working memory and/or attention tasks with means of functional magnetic resonance imaging (fMRI) or positron emission tomography (PET).
4. As contrasts used for fMRI or PET analysis we only included direct comparisons between attention or working memory task against a baseline condition for MS patients and healthy controls separately. Comparisons between healthy controls and MS patients without reporting brain activation for each group separately were not included.
5. Only studies reporting coordinates of a whole-brain analysis for patients and healthy controls separately were included. Studies reporting only results of regions of interest (ROI) analyses, volume of interest (VOI) analyses, or small volume correction (SVC) were excluded. Also, studies that reported only correlations of BOLD signal changes with respect to other measures were excluded.
6. All reported results had to be corrected for multiple testing at a significance level of *p* < 0.05, uncorrected data had to be thresholded at *p* < 0.005.
7. Included coordinates had to be reported in either standard Talairach space or the Montreal Neurologic Institute (MNI) space.

### 2.2 Activation likelihood estimation

Activation likelihood estimation (ALE) meta-analyses (Turkeltaub et al., 2002, 2012; Laird et al., 2005; Eickhoff et al., 2012), were performed using GingerALE 2.1 (www.brainmap.org/ale). If necessary, neuroanatomical coordinates reported in MNI space were transformed to Talairach space (Talairach and Tournoux, 1988) using icbm2tal transformation (Lancaster et al., 2007; Laird et al., 2010) implemented in GingerALE.

The ALE technique uses peak coordinates reported in functional neuroimaging studies as Gaussian probability distributions. The ALE algorithm is based on random-effects interference and controls for sample size by including the number of subjects in each study into calculation (Eickhoff et al., 2009). First, a whole-brain ALE map is created by estimating the likelihood of activation of each voxel. In the next step, the calculated ALE values are tested against the null hypothesis by using permutation testing (Eickhoff et al., 2012). The resulting statistical maps are thresholded at *p* < 0.05 and corrected for multiple testing using the false discovery rate (FDR). In the last step, GingerALE performs a cluster analysis based on the thresholded map with a minimum cluster size of 200 mm^3^. Separate meta-analyses were performed for patients and healthy controls. Finally, the resulting ALE maps for each group were subtracted from each other. Individual ALE maps were thresholded at a conservative level of *p* < 0.05 (FDR corrected), therefore a voxel-level threshold of *p* < 0.05 (uncorrected) was used for subtraction analyses to avoid inflating false negative results. To control for inordinate influence of one single study, further meta-analyses were performed, using a leave-one-out cross-validation procedure.

For visualization, whole-brain maps of thresholded ALE maps were imported into Multi-image analysis GUI (MANGO; http://ric.uthscsa.edu/mango) and overlaid onto a standardized anatomical template in Talairach space (colin1.1.nii; Laird et al., 2005).

## 3 Results

### 3.1 Literature review

Based on the systematic review of literature, a total of 42 articles that explored working memory and/or attention networks in MS using either fMRI or PET were identified. However, only nine studies in total, all using fMRI, fulfilled all inclusion criteria specified in the method section (see Table 1). These studies provided a total of 158 foci for healthy controls and 201 foci for patients with multiple sclerosis. Six of the included studies used correction for multiple testing at the peak- or cluster-level. The remaining three papers reported uncorrected *p*-value thresholds, the least conservative threshold was *p* < 0.005 uncorrected (one study).

**Fig. 1:**
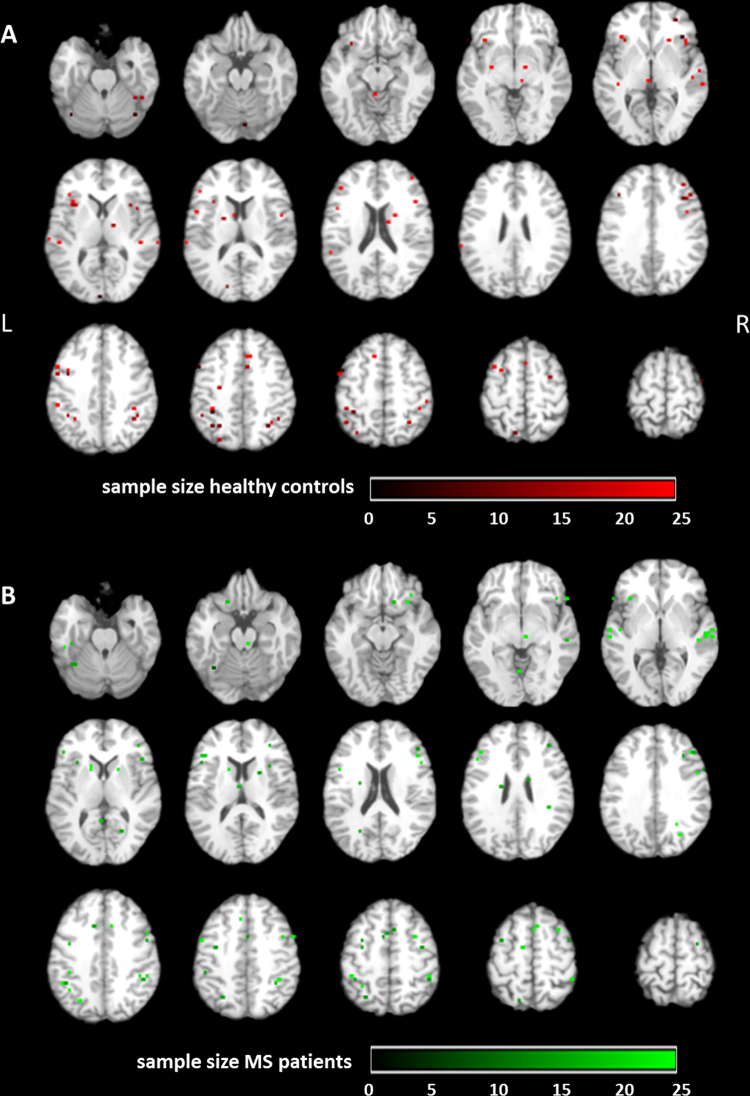
Visualization of all foci included in this ALE analysis, color-coded by sample size. Peak-voxels of included studies are projected on a standard anatomical template (colin1.1.nii) in axial orientation, referring to Talairach space. Voxel-size for all foci was set to 4x4x4 mm. Foci are reported for (A) healthy controls (red) and (B) MS patients (green) separately. (For interpretation of the references to color in this figure legend, the reader is referred to the web version of this article.)

For visualization of homogeneity of the included studies, all reported foci were presented in Talairach space for healthy controls and MS patients separately (Fig. 1A and B).

### 3.2 Significant ALE values for working memory and attention in healthy controls

**Fig. 2:**
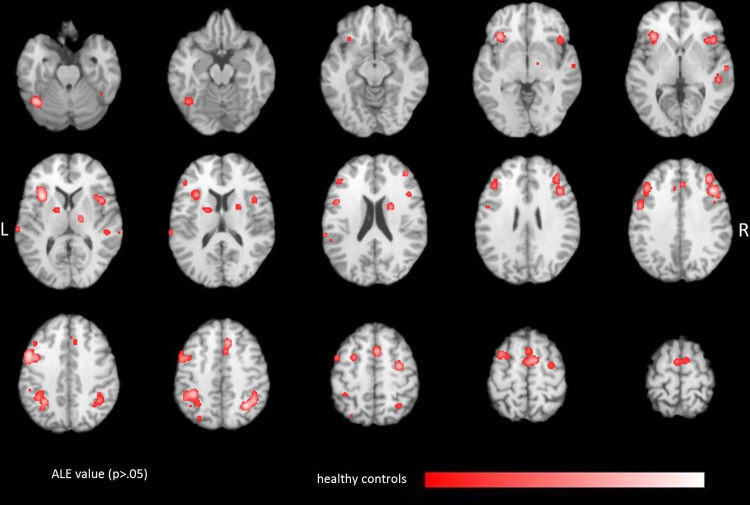
Localization of significant ALE values (*p* < 0.05, FDR corrected) due to attention and working memory tasks in healthy controls. ALE clusters are projected on a standard anatomical template (colin1.1.nii) in axial orientation, referring to Talairach space.

The ALE analysis of healthy subjects revealed 17 significant clusters for working memory and attention tasks (Table 2 and Fig. 2). We found significant ALE scores bilaterally in the dorsolateral and ventrolateral prefrontal cortex (DLPFC, VLPFC). ALE analysis further revealed significant clusters in the frontal eye field and the inferior parietal lobule, two areas responsible for visual attention. Moreover, significant ALE values were obtained in the insular cortex and in the thalamus.

### 3.3 Significant ALE values for working memory and attention in MS patients

**Fig. 3:**
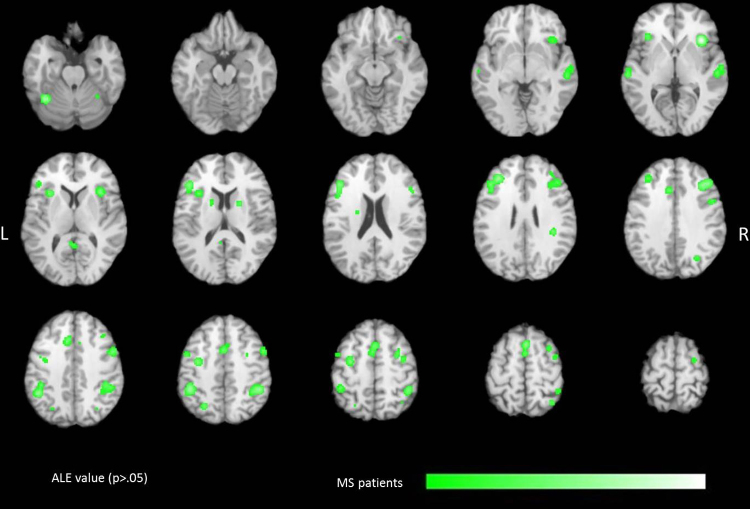
Localization of significant ALE values (*p* < 0.05, FDR corrected) due to attention and working memory tasks in MS patients. ALE clusters are projected on a standard anatomical template (colin1.1.nii) in axial orientation, referring to Talairach space.

For MS patients, ALE analysis obtained 24 statistically significant clusters related to working memory and attention tasks (Table 3 and Fig. 3). Similar to healthy controls, large significant clusters were found in the DLPFC and VLPFC, and in the inferior parietal lobule. Furthermore, ALE analysis revealed significant clusters in the superior and middle temporal gyri and in the insular cortex.

### 3.4 Comparison of ALE maps for healthy controls and MS patients

**Fig. 4:**
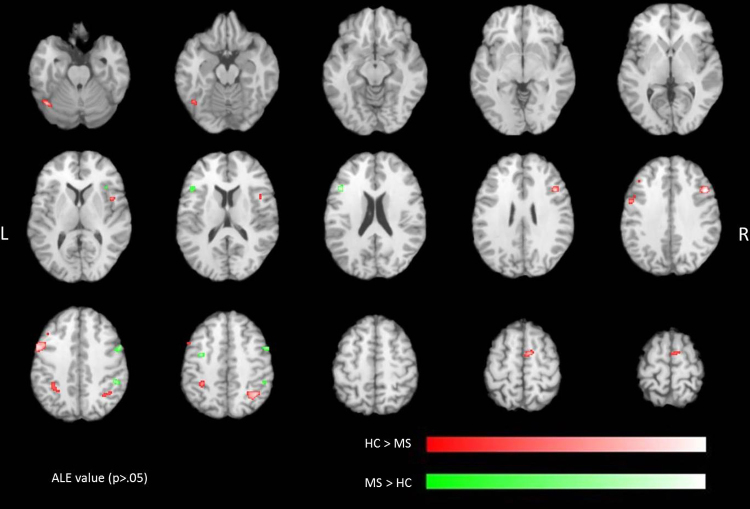
Significant ALE contrasts (*p* < 0.05, uncorrected) due to attention and working memory tasks in healthy controls versus MS patients (red) and in MS patients versus healthy controls (green). ALE clusters are projected on a standard anatomical template (colin1.1.nii) in axial orientation, referring to Talairach space. (For interpretation of the references to color in this figure legend, the reader is referred to the web version of this article.)

To investigate differences between MS patients and healthy controls, we calculated contrasts for the ALE maps of healthy controls versus MS patients and MS patients versus healthy controls. Significant ALE values related to higher likelihood of activation in healthy controls were found bilaterally in the inferior parietal lobule and the DLPFC as well as in the right VLPFC (Table 4 and Fig. 4). For the reverse contrast, indicating increased likelihood of activation in MS patients clusters were obtained in the left VLPFC and the right premotor area (Table 5 and Fig. 4).

### 3.5 Testing of robustness of ALE maps

**Fig. 5:**
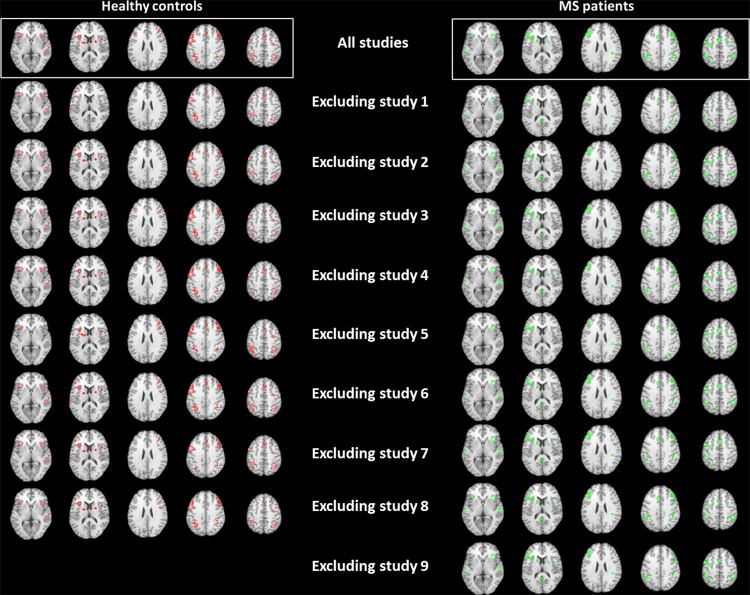
Significant ALE maps (*p* < 0.05, FDR corrected) for leave-one-out cross-validation procedure. ALE clusters are projected on a standard anatomical template (colin1.1.nii) in axial orientation, referring to Talairach space.

Due to the small number of included studies, one single study may influence resulting ALE maps excessively. Therefore, we performed nine additional meta-analyses, removing one single study from the data set. The results of this cross-validation procedure revealed high consistency of significant ALE maps (see Fig. 5).

## 4 Discussion

The aim of this review was to explore differences in brain activation between MS patients and healthy controls induced by working memory and attention tasks using the statistical power of a meta-analytic approach. Results of the ALE meta-analysis revealed the highest likelihood for activation in main attention and working memory related brain areas, such as the DLPFC and VLPFC and the inferior parietal lobule in healthy controls and MS patients. However, we found significantly increased activation bilaterally in the inferior parietal lobule and the DLPFC as well as the right VLPFC for healthy controls. In contrast, MS patients showed higher activation in the left VLPFC and the right premotor area.

Domains, typically impaired in MS patients are working memory, information processing speed and executive functions, including attention (for review see Lovera and Kovner, 2012). Additionally, the neuropsychological assessment of these abilities can easily be implemented in an fMRI setup so that we decided to only include fMRI studies investigating attention and working memory in this ALE meta-analysis. The included studies in MS patients involved fMRI paradigms such as the PASAT and the PVSAT, comprising working memory as well as attention abilities (Cardinal et al., 2008; Forn et al., 2008; Nagels et al., 2005). Although working memory and attention are distinguishable constructs, both processes are highly interactive (Chun and Turk-Browne, 2007; Gazzaley, 2011; Olivers et al., 2011; Burianová et al., 2012; Woodman et al., 2013). It is assumed that attention procedures actively participate in the manipulation and updating of working memory contents (for review see Awh et al., 2006). In different subtypes of MS especially PASAT served to detect and observe impairment of working memory and attention which have been identified among other cognitive deficits even at early stages of the disease (Deloire et al., 2005; Huijbregts et al., 2006). With large batteries of neuropsychological tests differences between cognitive impairment in PPMS and RRMS have been presented. Impaired information processing speed, attention, working memory, executive function, and verbal episodic memory have been identified in PPMS whereas in RRMS only information processing speed and working memory were impaired in comparison to healthy controls (Ruet et al., 2013). Restricted performance in working memory and attention related tasks has been indicated to be significantly associated with lesion load on structural MR images of the brain (Deloire et al., 2005).

Besides task-based functional imaging studies, patients with MS have increasingly been inspected using resting-state connectivity measures. Recently, systematic alterations of functional connectivity in resting-state networks have been identified in patients with MS. Characteristic modifications of functional connectivity at rest have been identified for the default mode network (Bonavita et al., 2011; Rocca et al., 2010), as well as for the sensorimotor network (Lowe et al., 2008), claiming changes already in very early stages of the disease (Faivre et al., 2012). Specific alterations of functional networks in patients with MS have been hypothesized to serve as an imaging biomarker for different cognitive functions, such as working memory (Sumowski et al., 2012) or attention (Loitfelder et al., 2012).

In this meta-analysis 17 significant ALE clusters were obtained for healthy controls. In contrast, the ALE analysis for MS patients revealed 24 significant clusters. The increased number of ALE clusters among MS patients might be explained by overreaching compensatory mechanisms, which have also been found for cognitive impairments in various diseases, such as major depression (Diener et al., 2012) or Alzheimer's disease (Browndyke et al., 2013). The results of this ALE meta-analysis revealed an increased likelihood of activation in the left VLPFC inducing an increased activation of the ventral attention network (VAN) compared to healthy controls, which showed more activation in the dorsal attention network (DAN). Findings of previous studies further point out the involvement of two different neural networks (Fox et al., 2006) in attention processes, which cover different components of attention (Corbetta et al., 2000; Hopfinger et al., 2000). The dorsal pathway is activated by expectation and anticipation, whereby top-down signals are transmitted to the sensory cortex (Hopfinger et al., 2000; Giesbrecht et al., 2003). In contrast, the ventral system is not pre-activated by expectation but plays an important role in reorienting attention based on new information (Serences et al., 2005; Shulman et al., 2009), reflecting a stimulus-driven bottom-up process (for review see Corbetta et al., 2008). Although both systems cover different aspects of attention, these networks interact in a systematic way (Kincade et al., 2005; Weissman and Prado, 2012; Wen et al., 2012). It is assumed that the interaction between the dorsal and the ventral system contributes to some sort of reorienting attention (for review see Corbetta et al., 2008). One reason for poorer performance of MS patients in the PASAT task may be a lack of preparatory expectation, reflected by an increased activation of the VAN in MS patients compared to healthy controls. Expectations based on pre-existing information contribute to simplification of decision by excluding unlikely events (Hopfinger et al., 2000; Astafiev et al., 2003). In MS patients, the DAN is less pre-activated by expectation, which may contribute to poorer performance in attention and working memory tasks.

Previous functional imaging studies revealed right hemisphere lateralization of the ventral pathway in healthy adults (Arrington et al., 2000; Corbetta et al., 2000; Downar et al., 2001; Fox et al., 2006). The results of the meta-analysis presented an increased likelihood of activation in the left VLPFC in the MS patient group compared to healthy controls, in which typical right hemisphere dominance was obtained. Atypical brain lateralization of cognitive functions has been detected in several neurological and psychiatric diseases such as autism (Lindell and Hudry, 2013), schizophrenia (Deep-Soboslay et al., 2010), or dyslexia (Leonard et al., 2006; for review see Rentería, 2012). It has been revealed that also anatomical differences in the human brain may indicate significant functional changes already in the fetus (Kasprian et al., 2011). Atypical lateralization of cognitive function may therefore potentially predict disease progression already in early stages.

### 4.1 Limitations

Although meta-analyses present a powerful method to calculate the statistical overlap between individual functional imaging studies, all data reducing approaches suffer from inherent drawbacks. The ALE technique, as all other meta-analysis techniques, is unable to assess subtle methodological differences in individual studies, or differences in preprocessing steps. However, it can be assumed that these potential errors do not systematically influence the results of a meta-analysis. In addition, sample size and number of reported foci are included into ALE algorithm (Eickhoff et al., 2009; Turkeltaub et al., 2012), therefore no individual study is able to bias the ALE analysis significantly (Turkeltaub et al., 2002). It should be recognized that meta-analyses are based upon previously published studies. However, studies without significant results or findings contradictory to the dominating opinion in a specific field of science may never be prepared for publication, what may cause a systematic overestimation of the results. MS patients usually show heterogeneous clinically symptoms, therefore, we defined strict inclusion criteria to create a data set, as homogeneous as possible. As a result, only nine studies fulfilled all criteria. Although the ALE algorithm controls for sample size of single studies and number of reported foci, calculations on a relatively small data set may result in increased influence of one single study on the results of the meta-analysis. Therefore, we monitored the impact of each study using a leave-one-out cross-validation procedure, in which no dominance of one single study was evident.

Multiple sclerosis is a complex and multi-layered disease with various disease specific influencing factors, such as age, disease type, and duration or type of medication, resulting in very inhomogeneous patient groups. Especially the age and the disease duration are correlated with the type of disease, as the SPMS type requires a longer duration until the onset of disease until it can be diagnosed. Therefore, generalized conclusions regarding disease progression based on findings of functional imaging studies are difficult. It has been shown that different aspects of cognition are impaired in different subtypes of MS (Ruet et al., 2013). Combining the results of studies including different phenotypes of MS is necessary in order to be able to analyze data cumulatively. FMRI data acquired in RRMS, RPMS, and SPMS were combined in the original studies as well as in our meta-analysis at the cost of sensitivity to differences in activation between these phenotypes.

### 4.2 Future directions

In multiple sclerosis the exact diagnosis, especially in early stages of the disease is challenging. Therefore the acquisition and combination of different indicators, such as lesion load, functional and structural information is of huge importance. However, in MS patient groups are typically inhomogeneous with respect to age, disease duration, or type of disease, therefore the development of new functional or structural biomarkers for diagnosis and disease progression is complicated. The aim of this meta-analysis was to summarize previous results of working memory and attention abilities in patients with MS to enable a general view on cognitive dysfunction in this disease. To gain more detailed insight into differences between disease subtypes concerning cognitive impairment and cognition related brain activation, studies including large patient groups of different subtypes are required. Furthermore, the resulting ALE maps will be provided online (http://www.meduniwien.ac.at/user/veronika.schoepf), and can be used as masks for further ROI analyses.

## Conflict of interest

The authors declare no conflict of interest in relation to this manuscript.

## Figures and Tables

**Table 1:** Neuroimaging studies of attention and working memory processes in multiple sclerosis.

Author, year	Healthy controls		Patients		Disease duration (y)	Type of disease	Cognitive paradigm	No of foci		Stereotactic space
	Age (years)	*N* (f/m)	Age (years)	*N* (f/m)				Controls	Patients	
Amann et al., 2011	33.9	15 (5/10)	37.6	15 (9/6)	5.9	RRMS	n-Back	39	39	TAL
Bonzano et al., 2009		18	32.5	23 (11/12)	6.9	RRMS	PVSAT	16	11	TAL
Cader et al., 2006	39.0	16 (10/6)	39.0	21 (15/6)	6.0	RRMS/RPMS	n-Back	8	8	MNI
Forn et al., 2007		10 (5/5)		17 (12/5)		RRMS	n-Back	10	10	TAL
Forn et al., 2006	31.1	10 (5/5)	32.7	15 (11/4)		RRMS	PASAT	8	14	TAL
Li et al., 2004	40.6	5	47.8	8			Auditory working memory task	6	2	TAL
Mainero et al., 2004		22 (11/11)	30.5	22 (14/8)	9.0	RRMS	PASAT/recall task	62	67	TAL
Penner et al., 2003		7	45.8	14 (13/1)	11.4	RRMS/SPMS	n-Back	9	22	TAL
Sumowski et al., 2010	−	−	43.8	18 (15/3)	9.5	RRMS/SPMS	n-Back	−	28	TAL

Total		103		153				158	201	

RRMS, relapsing-remitting multiple sclerosis; RPMS, relapsing-progressive multiple sclerosis; SPMS, secondary progressive multiple sclerosis; PASAT, paced auditory serial attention task; PVSAT, paced visual serial attention task.

**Table 2:** Significant FDR corrected ALE values for healthy controls.

Cluster number	Cluster volume (mm^3^)	ALE value	Talairach coordinates			Anatomical label
			*x*	*y*	*z*	
1	3448	0.02	−50	6	38	Middle Frontal Gyrus
		0.02	−40	2	38	Middle Frontal Gyrus
		0.01	−40	24	30	Middle Frontal Gyrus
		0.01	−48	2	48	Precentral Gyrus

2	2664	0.02	−32	14	8	Insular Cortex
		0.02	−34	22	−2	Inferior Frontal Gyurs

3	2432	0.02	42	20	30	Middle Frontal Gyrus
		0.02	38	34	30	Superior Frontal Gyrus
		0.01	40	42	20	Middle Frontal Gyrus

4	2392	0.02	−34	−42	42	Inferior Parietal Lobule
		0.02	−30	−52	38	Superior Parietal Lobule
		0.01	−32	−60	36	Angular Gyurs

5	1888	0.02	32	−58	44	Superior Parietal Lobule
		0.02	40	−52	42	Inferior Parietal Lobule
		0.01	38	−42	38	Supramarginal Gyrus

6	1464	0.02	8	22	42	Cingulate Gyrus
		0.02	4	10	50	Superior Frontal Gyrus

7	1240	0.02	10	0	56	Medial Frontal Gyrus
		0.01	0	−2	58	Medial Frontal Gyrus

8	1192	0.02	42	18	−2	Inferior Frontal Gyurs
		0.01	42	8	10	Insular Cortex
		0.01	34	16	2	Insular Cortex

9	1008	0.02	−40	−60	−22	Cerebellum (declive)
10	672	0.02	32	−8	50	Precentral Gyrus

11	632	0.01	−26	4	52	Middle Frontal Gyrus
		0.01	−34	6	56	Middle Frontal Gyrus

12	536	0.01	42	−50	−28	Cerebellum (culmen)
13	352	0.01	48	−32	4	Superior Temporal Gyrus
14	328	0.01	22	0	18	Lentiform Nucleus (putamen)
15	320	0.01	−16	−4	10	Lentiform Nucleus

16	264	0.01	−63	−30	10	Superior Temporal Gyrus
		0.01	−62	−34	16	Superior Temporal Gyrus

17	208	0.01	16	−14	8	Thalamus (ventral lateral nucleus)

**Table 3:** Significant FDR corrected ALE values for MS patients.

Cluster number	Cluster volume (mm^3^)	ALE value	Talairach coordinates			Anatomical label
			*x*	*y*	*z*	
1	2968	0.02	−32	34	26	Middle Frontal Gyrus
		0.02	−44	26	14	Inferior Frontal Gyrus
		0.02	−42	26	22	Middle Frontal Gyrus

2	2376	0.02	46	−44	44	Inferior Parietal Lobule

3	2016	0.02	−42	−42	44	Inferior Parietal Lobule
		0.02	−44	−48	38	Inferior Parietal Lobule

4	1896	0.02	2	14	54	Superior Frontal Gyrus
		0.01	0	8	44	Medial Frontal Gyrus
		0.01	0	2	52	Medial Frontal Gyrus

5	1616	0.02	42	28	30	Middle Frontal Gyrus
6	1592	0.03	34	18	2	Insular Cortex
7	1040	0.02	−36	−56	−26	Cerebellum (culmen)

8	976	0.02	55	−24	−2	Superior Temporal Gyrus
		0.02	60	−18	−2	Superior Temporal Gyrus

9	968	0.02	52	6	40	Middle Frontal Gyrus
10	944	0.02	−30	−8	46	Middle Frontal Gyrus
11	672	0.02	−8	18	34	Cingulate Gyrus
12	600	0.02	−30	16	10	Insular Cortex
13	576	0.02	32	−76	−32	Cerebellum (pyramis)
14	496	0.02	30	−54	−28	Cerebellum (anterior lobe)

15	400	0.01	32	8	52	Middle Frontal Gyrus
		0.01	30	2	46	Middle Frontal Gyrus

16	392	0.01	−59	−25	0	Superior Temporal Gyrus
		0.01	−58	−20	−1	Middle Temporal Gyrus

17	336	0.02	−34	−74	−36	Cerebellum (inferior semi-lunar lobule)
18	328	0.02	−24	−64	44	Superior Parietal Lobule
19	320	0.01	38	−4	50	Middle Frontal Gyrus
20	280	0.01	38	−34	24	Insular Cortex
21	240	0.01	−34	22	0	Insular Cortex
22	232	0.01	−46	2	46	Precentral Gyrus
23	216	0.01	20	2	12	Lentiform nucleus (putamen)

24	208	0.01	−4	−48	10	Posterior Cingulate Gyrus
		0.01	2	−52	8	Posterior Cingulate Gyrus

**Table 4:** Significant ALE values for the contrast healthy > patients.

Cluster number	Cluster volume (mm^3^)	ALE value	Talairach coordinates			Anatomical label
			*x*	*y*	*z*	
1	824	2.60	−53	5	36	Precentral Gyrus
		2.29	−44	8	38	Middle Frontal Gyrus

2	712	2.59	38	−58	40	Inferior Parietal Lobule
3	640	2.95	45	16	28	Middle Frontal Gyrus

4	352	2.19	6	−4	60	Medial Frontal Gyrus
		1.92	10	−2	56	Medial Frontal Gyrus

5	288	2.22	−42	−64	−22	Cerebellum (declive)
6	288	2.34	−32	−46	38	Inferior Parietal Lobule

**Table 5:** Significant ALE values for the contrast patients > healthy.

Cluster number	Cluster volume (mm^3^)	ALE value	Talairach coordinates			Anatomical label
			*x*	*y*	*z*	
1	320	2.66	−42	18	18	Middle Frontal Gyrus
		2.49	−46	18	20	Middle Frontal Gyrus

2	296	2.04	50	3	43	Middle Frontal Gyrus
		1.87	53	1	38	Precentral Gyrus
		1.76	45	3	40	Middle Fontal Gyrus
